# Associations between circulating resistin concentrations and left ventricular mass are not accounted for by effects on aortic stiffness or renal dysfunction

**DOI:** 10.1186/s12872-019-01319-w

**Published:** 2020-01-30

**Authors:** Glenda Norman, Gavin R. Norton, Vernice Peterson, Monica Gomes, Carlos D. Libhaber, Pinhas Sareli, Angela J. Woodiwiss

**Affiliations:** Cardiovascular Pathophysiology and Genomics Research Unit, School of Physiology, Faculty of Health Sciences, University of the Witwatersrand Medical School, 7 York Road, Parktown, Johannesburg, 2193 South Africa

**Keywords:** Obesity, Inappropriate left ventricular mass, Resistin, Aortic stiffness, Renal function

## Abstract

**Background:**

Although, in-part through an impact on left ventricular mass (LVM), resistin (an adipokine) may contribute to heart failure, whether this is explained by the adverse effects of resistin on aortic stiffness and renal function is unknown.

**Methods:**

Relationships between circulating resistin concentrations and LVM index (LVMI), and LVM beyond that predicted by stroke work (inappropriate LVM [LVM_inappr_]) (echocardiography) were determined in 647 randomly selected community participants, and in regression analysis, the extent to which these relations could be explained by aortic pulse wave velocity (PWV) or estimated glomerular filtration rate (eGFR) was evaluated.

**Results:**

Independent of confounders, resistin concentrations were independently associated with LVMI, LVM_inappr_, LV hypertrophy (LVH), PWV and eGFR. Furthermore, independent of confounders, LVMI, LVM_inappr_ and LVH were independently associated with PWV and eGFR. However, adjustments for either PWV or eGFR failed to modify the relationships between resistin concentrations and LVMI, LVM_inappr_ or LVH. Moreover, in multivariate regression analysis neither PWV nor eGFR significantly modified the contribution of resistin to LVM_inappr_ or LVMI.

**Conclusions:**

Independent relationships between circulating concentrations of the adipocytokine resistin and LVM are not explained by the impact of resistin on ventricular-vascular coupling or renal dysfunction. Resistin’s effects on LVM are therefore likely to be through direct actions on the myocardium.

## Background

Obesity is recognized as one of the major drivers of the increasing prevalence of cardiovascular disease worldwide. Importantly, obesity, independent of conventional risk factors and myocardial infarction, is a risk factor for several cardiovascular events including the progression to heart failure [1–3]. Insulin resistance may be a factor which mediates obesity-induced heart failure, however insulin resistance explains only a small percentage of these effects [2, 3]. Importantly, beyond adiposity indexes and insulin resistance, circulating concentrations of resistin (the adipocytokine expressed primarily in monocytes and macrophages, and which responds to inflammatory stimuli) [4–6] predict the progression to heart failure and its prognosis [7–12]. Thus, resistin may in-part explain independent relations between obesity and heart failure and circulating concentrations of resistin may act as potential biomarker for the development of heart failure in obesity. However, there is uncertainty as to the explanation that may account for the ability of circulating resistin concentrations to predict cardiac changes.

Recently, we reported the presence of strong, independent relations between circulating resistin concentrations and left ventricular mass (LVM) in a large community-based sample [13]. As LVM is a well-recognized determinant of the progression to heart failure, the impact of resistin may in-part be explained by effects on LVM. Relationships between resistin and LVM may nevertheless be accounted for by several mechanisms. In this regard, effects on LVM may be through direct actions on the myocardium as cardiomyocyte overexpression of resistin promotes myocardial hypertrophy in mice [14–17]. However, resistin may also mediate increases in LVM through indirect actions. Indeed, resistin independently associates with decreases in glomerular function [18] and increases in LVM are strongly associated with renal dysfunction beyond hemodynamic effects [19]. Alternatively, beyond blood pressure effects, resistin may also enhance afterload to the LV through ventricular-vascular coupling. Ventricular-vascular coupling may account for resistin’s effect on LVM through an enhanced aortic stiffness [20], as aortic stiffness associates with LVM beyond central arterial blood pressure [21]. As it is uncertain whether associations between circulating resistin concentrations and adverse cardiac effects may be accounted for by indirect (renal dysfunction or ventricular-vascular coupling) or direct effects, in the present study we assessed the extent to which independent relationships between circulating resistin concentrations and LVM in a large community-based sample are explained by an impact of resistin on renal or aortic function. Furthermore, as general inflammation (as indexed by circulating C-reactive protein [CRP]) may in-part explain the relationship between aortic stiffness and LVM (ventricular-vascular coupling) in hypertensive patients with metabolic syndrome [22], we assessed whether relationships between circulating resistin concentrations and LVM in a large community-based sample are independent of CRP.

## Methods

### Study group

The protocol for the present study was approved by the Committee for Research on Human Subjects of the University of the Witwatersrand (approval numbers: M02–04-72 renewed as M07–04-69, M12–04-108 and M17–01-01). Informed, written consent was given by all participants and the study was carried out in accordance with the principles outlined in the Helsinki declaration. The design of the current study has been described previously [13, 23, 24]. Of the 1024 participants from nuclear families of black African descent (with siblings older than 16 years of age) randomly recruited from the South West Township (SOWETO) of Johannesburg, South Africa, in a sub-study 647 participants had echocardiography, aortic pulse wave velocity and measurements of plasma resistin concentrations. Of the sample 487 were not receiving anti-hypertensive treatment.

### Clinical, demographic, anthropometric and blood measurements

A standardized questionnaire was used to obtain demographic and clinical data [23, 24]. Measurements of indices of obesity were made using standard approaches as previously described [13, 23, 24]. Standard laboratory blood tests were performed and diabetes mellitus (DM) or abnormal blood glucose control was defined as previously described [13]. A trained nurse-technician obtained brachial blood pressure (BP) measurements using a standard mercury sphygmomanometer. Metabolic syndrome was defined as a combination of the presence of WC ≥88 cm in women and ≥ 102 cm in men, fasting blood glucose ≥5.6 mmol/l, triglycerides ≥1.7 mmol/l, HDL cholesterol< 1.04 mmol/l in men and < 1.30 mmol/l in women, and systolic BP ≥130 or diastolic BP ≥85 or treatment for hypertension.

Serum creatinine concentrations were measured using the Advia Chemistry systems (Siemens) with calibration traceable to isotope dilution mass spectrometry (IDMS). The 4-varible CKD-EPI equation was employed to estimate glomerular filtration rate (GFR) [25]. Blood samples for the measurement of insulin and resistin concentrations were centrifuged and immediately stored at -80 °C. Concentrations of fasting plasma insulin, resistin and C-reactive protein (CRP) were measured using a chemiluminescent immunometric assay (insulin) and enzyme-linked immunosorbent assays (resistin and CRP), and insulin resistance estimated by the homeostasis model assessment of insulin resistance (HOMA-IR) as previously described [13].

### Echocardiography

Echocardiography was performed as described previously [13, 23, 24, 26]. A standard formula was used to determine left ventricular mass [27] and indexed (LVMI) to height^2.7^. Left ventricular hypertrophy was defined as an LVMI> 47 g/m^2.7^ in women and LVMI> 50 g/m^2.7^ in men [28]. Using the Z-derived method, stroke volume was calculated from the difference between LV end diastolic and systolic volumes [26]. To determine the impact of resistin on LVM beyond work load, we determined inappropriate LVM (LVM_inappr_). The extent of LVM_inappr_ was established from predicted LVM as previously described [13, 26]. Relative wall thickness was determined as (LV septal wall thickness in diastole + LV posterior wall thickness in diastole) / (LV end diastolic diameter in diastole). Left atrial volume indexed to body surface area, was calculated using the area-length method.

### Aortic stiffness

Aortic stiffness was assessed using carotid-femoral (aortic) pulse wave velocity (PWV) as previously described [20, 21], as aortic stiffness associates with LVM beyond central arterial blood pressure. In this regard, after participants had rested for 15 min in the supine position, sequential arterial waveforms at the carotid and femoral pulse were recorded by applanation tonometry during an 8-s period each using a high-fidelity SPC-301 micromanometer (Millar Instrument, Inc., Houston, Texas) interfaced with a computer employing SphygmoCor, version 9.0 software (AtCor Medical Pty. Ltd., West Ryde, New South Wales, Australia). The time delay in the pulse waves between the carotid and femoral sites was determined using an electrocardiograph-derived R wave as a fiducial point. Pulse transit time was obtained from the average of 10 consecutive beats. The distance which the pulse wave travels was determined as the difference between the distance from the femoral sampling site to the suprasternal notch, and the distance from the carotid sampling site to the suprasternal notch.

### Statistical analyses

SAS software, version 9.4 (SAS Institute Inc., Cary, NC, USA) was used for database management and statistical analyses. Continuous data are represented as mean ± SD. Comparisons of unadjusted means and proportions were made using the large-sample z-test and the χ^2^-statistic, respectively. Log transformation of resistin concentrations was performed as data were non-normally distributed and transformation improved distribution [20]. For continuous data, bivariate correlations were assessed using Pearson’s correlation coefficients. Multivariate linear regression analysis with appropriate adjustors was used to assess independent relationships. To determine the impact of eGFR or aortic PWV on the contribution of circulating resistin concentrations to LVM, multivariate regression analysis was performed. Probability values were further adjusted for non-independence of family members as previously described [13].

## Results

### Participant characteristics

A high proportion of participants was overweight or obese and had central (abdominal) obesity (Table 1). In addition, a high percentage of participants had hypertension (Table 1). However, only 24.4% of participants had 3 or more components of the metabolic syndrome (Table 1). LVH was present in 26.3% of participants.

**Table 1:** Characteristics of study participants

	All Participants
	(*n* = 647)
% Women	62.8
Age (years)	44.5 ± 18.2
Body mass index (kg/m^2^)	28.9 ± 7.5
Waist circumference (cm)	90.3 ± 16.3
% Overweight/obese	27.0/40.7
% Central obesity	42.5
% Hypertension	47.4
% Treated for DM	8.6
% DM or HbA1c > 6.5%	14.1
% Regular smoking	15.8
% Regular alcohol	20.2
% Metabolic syndrome^a^	28.9/24.7/14.1/7.7/2.6
Office SBP/DBP (mm Hg)	128 ± 22/83 ± 12
LV mass (LVM)(g)	151 ± 52
LVM index (g/m^2.7^)	41.6 ± 14.9
% LVH	26.3
% Actual LVM/Predicted LVM	133 ± 36
Actual LVM-predicted LVM (g)	37.2 ± 39.2
Aortic PWV (m/sec)	6.10 ± 2.76
eGFR (mls/min/1.73m^2^)	94.8 ± 21.7
Resistin (ng/ml)	10.5 (range = 1.4 to 83.0)
C-reactive protein (ng/ml)	3.64 (range = 0.01 to 71.97)

Continuous data are expressed as mean ± SD or median (range). ^a^Metabolic syndrome is percentage of individuals with 1/2/3/4/5 components of metabolic syndrome. *DM* diabetes mellitus, *SBP* systolic blood pressure, *DBP* diastolic BP, *PWV* pulse wave velocity, *eGFR* estimated glomerular filtration rate

### Associations between resistin concentrations and metabolic abnormalities

On bivariate analysis resistin was related to BMI and WC, but not to HOMA-IR (Additional file 1: Table S1). With adjustments for age, sex, regular smoking, regular alcohol intake and treatment for hypertension, BMI (partial *r* = 0.03, *p* = 0.40) and WC (partial *r* = 0.02, *p* = 0.59) were not independently associated with resistin concentrations. Similarly, no independent associations between adiposity indexes and resistin concentrations were noted in those not receiving antihypertensive therapy (BMI: partial *r* = 0.09, *p* = 0.06; WC; partial *r* = 0.07, *p* = 0.11). No independent relationships were noted between resistin concentrations and HOMA-IR (partial *r* = − 0.009, *p* = 0.82).

### Associations between resistin concentrations and eGFR, aortic stiffness or CRP

On bivariate analysis resistin was related to eGFR, PWV and CRP (Additional file 1: Table S1). With adjustments for age, sex, BMI, regular smoking, regular alcohol intake, treatment for hypertension, systolic, diastolic or mean arterial blood pressure, the presence of diabetes mellitus or an HbA1c > 6.5%, circulating resistin concentrations were independently associated with eGFR (partial *r* = − 0.20, *p* < 0.0001). With the same adjustments, circulating resistin concentrations were also independently associated with aortic PWV (partial *r* = 0.080, *p* < 0.05) and with CRP (partial *r* = 0.19, *p* < 0.0001).

### Associations between eGFR, aortic stiffness or CRP and LVM

With adjustments for age, sex, body weight, regular smoking, regular alcohol intake, treatment for hypertension, systolic blood pressure, the presence of diabetes mellitus or an HbA1c > 6.5%, eGFR was independently associated with LVM_inappr_ (Table 2), LVMI (Table 3), and LVH (Odds ratio (95% CI) = 0.985 (0.973 to 0.998, *p* < 0.02). With the same adjustments, aortic PWV was similarly independently associated with LVMI (Table 3) and LVH (Odds ratio (95% CI) = 1.107 (1.014 to 1.208, *p* < 0.02), but not with LVM_inappr_ (Table 2). However, with the same adjustments, CRP was not independently associated with LVM_inappr_ (Table 2), LVMI (Table 3), or LVH (Odds ratio (95% CI) = 1.191 (0.764 to 1.855, *p* = 0.44).

**Table 2:** Impact of adjustments for estimated glomerular filtration rate (eGFR), pulse wave velocity (PWV) or C-reactive protein (CRP) on the relative contribution of circulating resistin concentrations to variations in inappropriate left ventricular mass (LVM_inappr_) in a community sample (*n* = 647)

Models with→	^a^		^a^ + eGFR		^a^ + PWV		^a^ + CRP	
	β-coeff ±SEM	*p* value	β-coeff±SEM	*p* value	β-coeff±SEM	*p* value	β-coeff±SEM	*p* value
Age	−0.022 ± 0.051	=0.67	−0.118 ± 0.060	=0.048	−0.022 ± 0.055	=0.69	−0.034 ± 0.051	=0.50
Male gender	0.091 ± 0.045	=0.037	0.090 ± 0.044	=0.043	0.091 ± 0.044	=0.047	0.091 ± 0.045	=0.042
BMI	0.244 ± 0.046	< 0.0001	0.227 ± 0.046	< 0.0001	0.257 ± 0.049	< 0.0001	0.216 ± 0.049	< 0.0001
SBP	−0.088 ± 0.043	=0.0075	−0.133 ± 0.043	=0.0022	−0.089 ± 0.046	=0.0076	−0.122 ± 0.043	=0.0050
Log resistin	0.119 ± 0.038	=0.0018	0.130 ± 0.038	=0.0006	0.118 ± 0.038	=0.0020	0.149 ± 0.038	=0.0001
eGFR or PWV or CRP	–	–	−0.143 ± 0.053	=0.0069	0.002 ± 0.050	=0.98	0.012 ± 0.045	=0.78
HOMA-IR	0.114 ± 0.039	0.0035	0.098 ± 0.039	=0.012	0.083 ± 0.037	=0.0027	0.121 ± 0.039	=0.0019

*β-coeff* standardized β-coefficient, *BMI* body mass index, *SBP* office systolic blood pressure; *HOMA-IR* homeostasis model of insulin resistance. ^a^Additional factors included in the stepwise regression models include regular smoking, regular alcohol intake, treatment for hypertension and diabetes mellitus or an HbA1c > 6.5%

**Table 3:** Impact of adjustments for estimated glomerular filtration rate (eGFR), aortic pulse wave velocity (PWV) or C-reactive protein (CRP) on the relative contribution of circulating resistin concentrations to variations in left ventricular mass index (LVMI) in a community sample (*n* = 647)

Models with→	^a^		^a^ + eGFR		^a^ + PWV		^a^ + CRP	
	β-coeff ±SEM	*p* value	β-coeff±SEM	*p* value	β-coeff±SEM	*p* value	β-coeff±SEM	*p* value
Age	0.156 ± 0.048	=0.0012	0.097 ± 0.056	=0.09	0.111 ± 0.052	=0.028	0.155 ± 0.049	=0.0015
Male gender	0.030 ± 0.040	=0.45	0.032 ± 0.040	=0.42	0.032 ± 0.040	=0.43	0.029 ± 0.041	=0.47
Weight	0.147 ± 0.039	=0.0002	0.148 ± 0.039	=0.0002	0.153 ± 0.039	=0.0001	0.150 ± 0.041	=0.0002
SBP	0.218 ± 0.041	< 0.0001	0.211 ± 0.041	< 0.0001	0.191 ± 0.043	< 0.0001	0.212 ± 0.041	< 0.0001
Log resistin	0.090 ± 0.036	=0.014	0.075 ± 0.036	=0.044	0.084 ± 0.036	=0.022	0.087 ± 0.036	=0.017
eGFR or PWV or CRP	–	–	−0.098 ± 0.051	=0.052	0.102 ± 0.048	=0.047	0.004 ± 0.043	=0.92
HOMA-IR	0.094 ± 0.037	=0.012	0.082 ± 0.038	=0.032	0.090 ± 0.037	=0.017	0.096 ± 0.037	=0.011

*β-coeff* standardized β-coefficient, *BMI* body mass index, *SBP* office systolic blood pressure, *HOMA-IR* homeostasis model of insulin resistance. ^a^Additional factors included in the stepwise regression models include regular smoking, regular alcohol intake, treatment for hypertension and diabetes mellitus or an HbA1c > 6.5%

### Independent relationships between resistin concentrations and LV dimensions

**Fig. 1:**
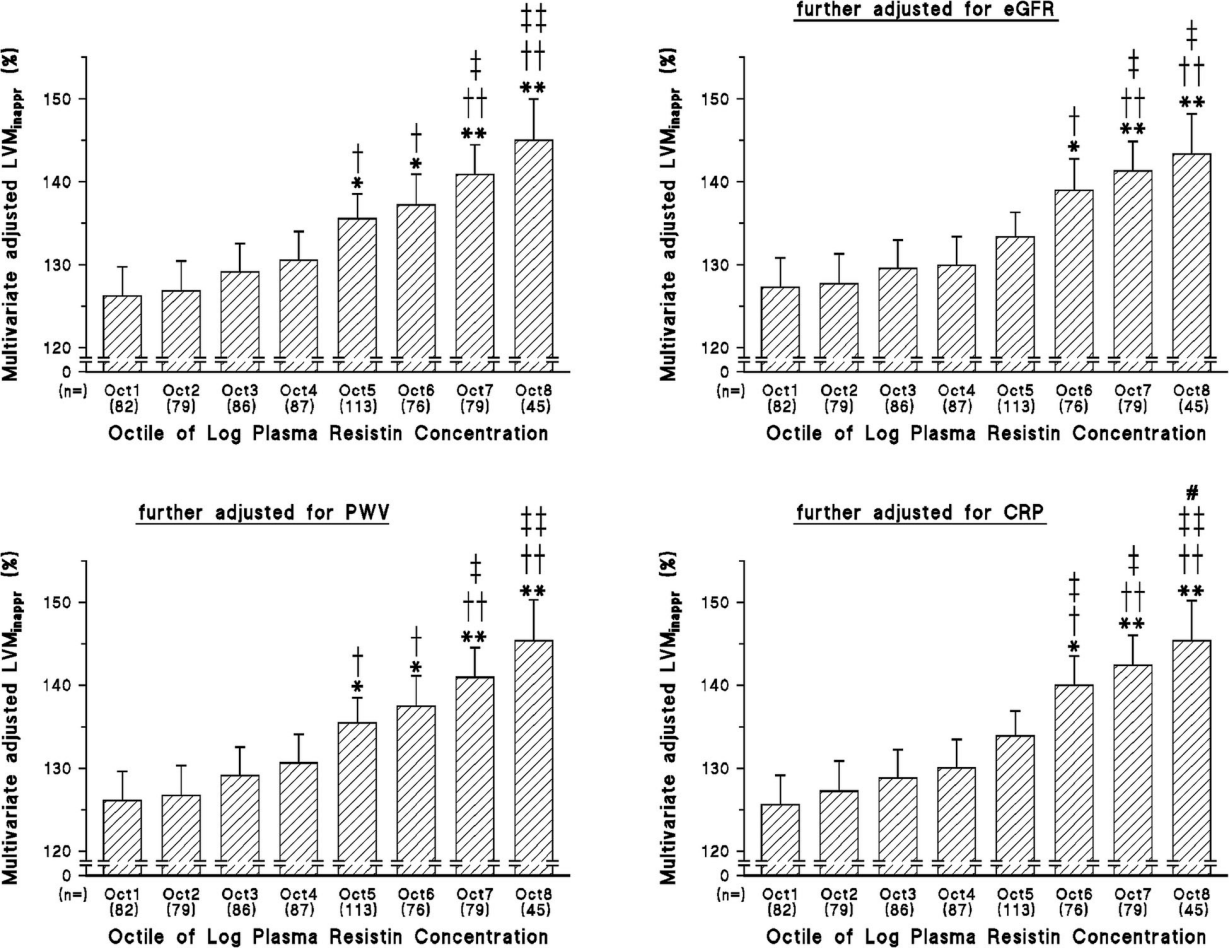
Impact of adjustments for aortic pulse wave velocity (PWV), estimated glomerular filtration rate (eGFR) or C-reactive protein (CRP) on multivariate adjusted left ventricular mass (LVM) beyond that predicted by stroke work (inappropriate LVM or LVM_inappr_) across octiles (Oct) of log resistin concentrations. Additional adjustments are for age, sex, body mass index, office systolic blood pressure, treatment for hypertension, diabetes mellitus or an HbA1c > 6.5%, regular smoking, and regular alcohol intake. *p < 0.05, **p < 0.005 vs octile 1; ^†^*p* < 0.05, ^††^*p* < 0.005 vs octile 2; ^‡^*p* < 0.05, ^‡‡^*p* < 0.005 vs octiles 3 and 4; #*p* < 0.05 vs octile 5

On bivariate analysis circulating resistin concentrations, were associated with LVMI (*p* < 0.0001), LVM_inappr_ (*p* < 0.0001) and relative wall thickness (*p* < 0.02), but not left atrial volume index (*p* = 0.09). In stepwise regression models BMI, WC, HOMA-IR, and resistin concentrations were all directly and independently associated with LVM_inappr_ (Tables 2 and Additional file 1: Table S2) and LVMI (Tables 3 and Additional file 1: Table S3), but not with relative wall thickness (*p* = 0.14) or left atrial volume index (*p* = 0.26). The relationships with LVMI and LVM_inappr_ were not affected by the inclusion of either eGFR, aortic PWV or CRP in the models (Tables 2, 3, Additional file 1: Table S2 and Table S3). LVM_inappr_ increased across octiles of resistin concentrations independent of adjustments including BMI (Fig. 1). LVM_inappr_ was noticeably larger in the highest 2 octiles of resistin concentrations in comparison to the lowest 2 octiles of resistin concentrations (Fig. 1). These relationships were similarly unaffected by adjustments for either eGFR, aortic PWV or CRP (Fig. 1). With adjustments including WC, LVM_inappr_ similarly increased across octiles of resistin concentrations (Additional file 1: Figure S1), and these relationships were unaffected by adjustments for either eGFR, aortic PWV or CRP (Additional file 1: Figure S1).

### Independent relationships between resistin concentrations and LVH

**Fig. 2:**
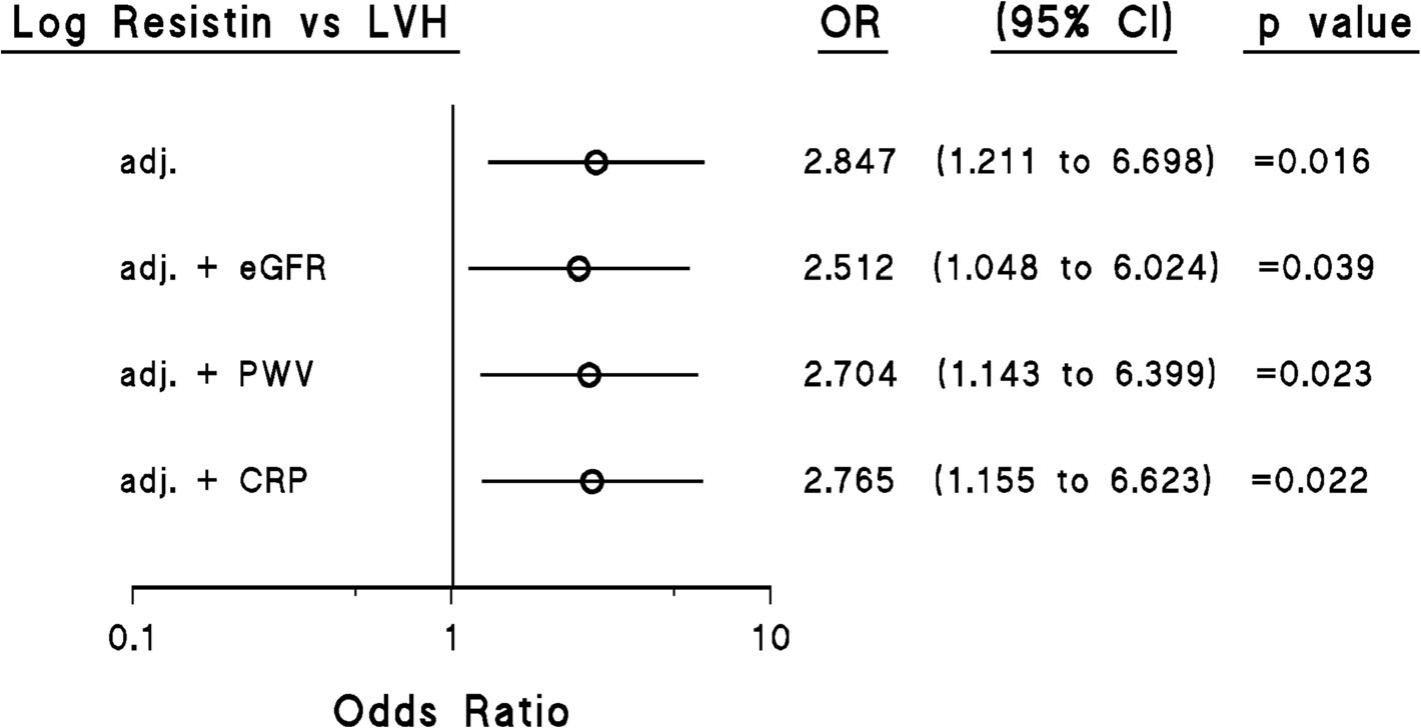
Impact of adjustments for aortic pulse wave velocity (PWV), estimated glomerular filtration rate (eGFR) or C-reactive protein (CRP) on multivariate adjusted relations between log circulating resistin concentrations and left ventricular hypertrophy (LVH) in a community sample (*n* = 170 with LVH). Additional adjustments are for age, sex, body mass index, systolic blood pressure, treatment for hypertension, diabetes mellitus or an HbA1c > 6.5%, regular smoking, and regular alcohol intake

Resistin concentrations were also associated with LVH independent of confounders including age, sex, BMI, SBP, regular smoking, regular alcohol consumption, treatment for hypertension, diabetes mellitus or an HbA1c > 6.5% and log HOMA-IR (Fig. 2). Importantly, these relationships were not affected by the inclusion of either eGFR, aortic PWV or CRP in the models (Fig. 2). When waist circumference was included as a confounder instead of BMI, resistin concentrations were also independently associated with LVH (Additional file 1: Figure S2), and these relationships were not affected by the inclusion of either eGFR, aortic PWV or CRP in the models (Additional file 1: Figure S2).

### Contribution of resistin concentrations to LVM

In multivariate regression analyses, resistin concentration was second to BMI as a determinant of LVM_inappr_ and contributed as much as SBP to LVM_inappr_ (Table 4). For LVMI the contribution of resistin concentration was similar to HOMA-IR, but less that age, SBP and weight (Table 4). The inclusion of either PWV, eGFR or CRP in the models had no impact on the contribution of resistin to LVM_inappr_ (partial r^2^ with PWV in the model = 0.022, *p* < 0.0001; partial r^2^ with eGFR in the model = 0.022, *p* < 0.0001; partial r^2^ with CRP in the model = 0.027, *p* < 0.0001). The contribution of resistin to LVMI was not modified by the inclusion of PWV or CRP in the model (partial r^2^ with PWV in the model = 0.009, *p* < 0.01; partial r^2^ with CRP in the model = 0.010, *p* < 0.005), and only marginally modified by the inclusion of eGFR in the model (partial r^2^ = 0.005, *p* < 0.05). In the models including waist circumference (Additional file 1: Table S4), resistin concentration contributed as much as SBP to LVM_inappr_ and for LVMI the contribution of resistin concentration was similar to age, but less than waist circumference and SBP. The contribution of resistin to LVM_inappr_ was not modified by the inclusion of either eGFR or CRP in the models (partial *r*^2^ with eGFR in the model = 0.030, *p* < 0.0001; partial *r*^2^ with CRP in the model = 0.030, *p* < 0.0001), and only marginally modified by the inclusion of PWV in the model (partial r^2^ with PWV in the model = 0.021, *p* = 0.0001). The contribution of resistin to LVMI was not modified by the inclusion of PWV or CRP in the model (partial *r*^2^ with PWV in the model = 0.009, *p* < 0.01; partial *r*^2^ with CRP in the model = 0.012, *p* = 0.001), and only marginally modified by the inclusion of eGFR in the model (partial *r*^2^ = 0.007, *p* < 0.02).

**Table 4:** Circulating resistin concentrations as a determinant of inappropriate left ventricular mass (LVM_inappr_) or left ventricular mass index (LVMI) in a community sample (n = 647)

Models with→	LVM_inappr_			LVMI	
	^a^			^a^	
	Partial r^2^	*p* value		Partial r^2^	*p* value
BMI	0.081	< 0.0001	Age	0.128	< 0.0001
Log resistin	0.022	< 0.0001	SBP	0.037	< 0.0001
SBP	0.019	=0.0002	Weight	0.028	< 0.0001
HOMA-IR	0.014	=0.0015	Log resistin	0.009	=0.010
Male gender	0.007	=0.020	HOMA-IR	0.008	=0.015
Age	0.0004	=0.57	Male gender	0.0008	=0.41
Model r^2^	0.144		Model r^2^	0.214	

*BMI* body mass index, *SBP* office systolic blood pressure, *HOMA-IR* homeostasis model of insulin resistance. ^a^Additional factors included in the stepwise regression models include regular smoking, regular alcohol intake, treatment for hypertension and diabetes mellitus or an HbA1c > 6.5%

### Contribution of PWV or eGFR to resistin-related increases in LVM

In mediation analysis, the contribution of PWV to resistin-related increases in LVM and LVM_inappr_ was negligible (0.37 ± 0.09 g/m^2.7^ and − 0.06 ± 0.23% respectively). Similarly, the contribution of eGFR to resistin-related increases in LVM and LVM_inappr_ was negligible (0.93 ± 0.09 g/m^2.7^ and 3.28 ± 0.23% respectively. When waist circumference (instead of body weight or BMI) was included in the multivariate model, the contributions of PWV or eGFR to resistin-related increases in LVM and LVM_inappr_ were also negligible (PWV: 0.29 ± 0.09 g/m^2.7^ and − 0.14 ± 0.23% respectively; eGFR: 0.94 ± 0.09 g/m^2.7^ and 3.05 ± 0.23% respectively).

## Discussion

The main findings of the current study are as follows: In a large, randomly selected, community-based sample, circulating resistin concentrations were independently associated with indexes of structural changes in the LV including LVMI, LVM_inapp_, and LVH, as well as with both eGFR and aortic stiffness (carotid-femoral PWV). In this regard, consistent with the well recognized impact of ventricular-vascular coupling and renal dysfunction on LVM, both eGFR and PWV were also independently associated with LVM and LVH. However, in multivariate regression, neither eGFR nor aortic PWV could account for independent relationships between circulating resistin concentrations and LVM or LVH. In addition, in multivariate regression analysis neither PWV nor eGFR significantly modified the contribution of resistin to LVM_inappr_ or LVMI.

The mechanisms that may explain the ability of circulating resistin concentrations to predict the progression to heart failure and its prognosis [7–12] are uncertain. Whilst overexpression of resistin mediates several adverse effects on the myocardium in mice [15, 16], resistin may produce several alternative changes that may mediate increases in LVM through a multitude of indirect mechanisms. In this regard, resistin was originally identified as a molecule with the ability to mediate insulin resistance in mice and insulin resistance in obesity is a well-recognized cause of cardiac hypertrophy and dysfunction. However, the role of resistin in mediating insulin resistance in humans is unclear and as we have previously described [13] and similarly demonstrated in the present study, resistin is not associated with HOMA-IR. Moreover, HOMA-IR does not explain independent relationships between resistin and LVM [13]. Thus, an impact of resistin on LVM through direct myocardial effects mediated by inflammatory changes [4–6], requires consideration. Nevertheless, several alternative indirect effects of resistin need to be excluded. In the present study we addressed the possibility that two important effects of resistin (increases in aortic stiffness or renal dysfunction) may explain the adverse cardiac actions of resistin.

Increases in aortic stiffness are strongly associated with LVM [21, 29] and these relationships are independent of brachial and aortic pulse pressure as well as the aortic wave component that is driven by increases in aortic stiffness (forward wave pressure) [21]. In this regard, stiffness of the proximal aorta is thought to increase afterload to the left ventricle through ventricular-vascular coupling [29]. As resistin is independently associated with aortic PWV but neither aortic pulse pressure, nor the forward wave pressure [20], the possibility that resistin may mediate increases in LVM through ventricular-vascular coupling requires consideration. Importantly, in the present study, as previously described [20, 21], circulating resistin concentrations were independently associated with aortic PWV and PWV was independently associated with LVM. However, adjustments for PWV failed to influence resistin-LVM relations, and in multivariate regression analysis PWV failed to modify the contribution of resistin to LVM_inappr_ or LVMI. Thus, together with the fact that resistin concentrations are not independently associated with brachial, central aortic or ambulatory BP [20], independent relations between resistin concentrations and LVM are unlikely to be accounted for by load-dependent effects, including an effect of resistin on aortic stiffness. Modifying afterload to the left ventricle is thus not a therapeutic option in preventing the adverse effects of resistin on the heart.

Through renal dysfunction, well before the onset of renal failure, reductions in estimated glomerular filtration rate are strongly associated with LVM and these effects are not attributed to an impact of hemodynamic factors [19]. As resistin is a well-recognized determinant of renal damage [18], and in the present study was independently associated with a decrease in eGFR, the possibility that resistin mediates increases in LVM through effects on renal function was considered. Importantly however, although eGFR was indeed strongly and independently associated with LVM, adjustments for eGFR did not modify resistin-LVM relations. In addition, in multivariate regression analysis eGFR failed to modify the contribution of resistin to LVM_inappr_ and only had a marginal impact on the contribution of resistin to LVMI. Thus, in risk-prediction, the assessment of eGFR, a simple and cost-effective assessment of cardiovascular risk, will not adequately identify the adverse effects of resistin on the LV.

Resistin is induced in response to various pro-inflammatory stimuli such as tumor necrosis factor-α (TNF-α), interleukin (IL)-6, and IL-1β [4–6]. Furthermore, resistin has been shown to up-regulate the expression of pro-inflammatory cytokines TNF-α, IL-6, IL-12, and monocyte chemoattractant protein-1 in macrophages. This raises the question of whether the independent relationships between resistin and LVM can be attributed to general inflammatory effects. In this regard however, whilst resistin is independently associated with LVM, the general inflammatory molecule C-reactive protein is not [13]. Furthermore, although circulating concentrations of TNF-α, and IL-6 independently associate with concentric LV remodeling, there is no relationship between these molecules and LVM [30]. Although C-reactive protein may in-part explain ventricular-vascular coupling [22], the relationships between resistin and LVM in the present study were independent of C-reactive protein. Hence, it is unlikely that the resistin-LVM relationships can be explained by general inflammatory effects. Whether resistin has local effects on macrophage function in the myocardium is nevertheless possible. Importantly however, these effects are clearly indexed by circulating resistin concentrations, thus supporting the view that circulating resistin concentrations may be employed as a biomarker of the progression to heart failure.

The relationships between circulating resistin concentrations and LVM beyond indices of obesity (BMI or waist circumference) despite the high proportion of participants with obesity in the current study, suggest that the adverse effects of resistin on LVM are through effects beyond adipose tissue. However, unlike mice, resistin in humans is often undetectable in adipocytes, and the major source of circulating resistin is likely to be peripheral blood mononuclear cells [6]. Indeed, obese individuals, who are likely to have a greater infiltration of macrophages in adipose tissue, show increased expression of resistin in adipose tissue samples and higher circulating concentrations of resistin than lean individuals [31]. Importantly, the lack of independent relationships between adiposity indexes and circulating resistin concentrations in the present study are not attributed to inaccuracies in measures of excess adiposity as expected relationships between adiposity indexes and LVM were noted. Consequently, although the impacts of resistin on LVM described in the current study are indeed independent of indexes of either general (BMI) or central (abdominal, waist circumference) obesity, it is possible that the circulating resistin concentrations may in some way be associated with alterations in adipose tissue. The exact adipose tissue changes responsible for the resistin effects on LVM however require further study.

There are several limitations to the current study that require consideration. In this regard, the present study was cross-sectional in design and hence no conclusions regarding causality can be drawn. However, as previously emphasized [13], the present results are supported by extensive preclinical findings suggesting a myocardial hypertrophic effect of resistin [14–17]. Second, the present study was conducted in one ethnic group in a specific community selected because of the high prevalence of obesity, and the limited confounding effects of antihypertensives on LVM [13]. Hence, whether resistin-LVM relationships are consistent across populations, is uncertain.

## Conclusions

In the current study performed in a large sample of community participants in whom a high proportion had obesity, we show that despite striking relations noted between circulating resistin concentrations and both aortic stiffness and glomerular function, these effects do not account for the independent relationships between circulating resistin concentrations and LVM or LVH. Thus, neither ventricular-vascular coupling nor renal dysfunction is likely to explain the relationships between circulating resistin concentrations and LV hypertrophy. Circulating resistin concentrations may therefore be a biomarker of direct rather than indirect actions of resistin on the myocardium which may ultimately predict the progression to heart failure.

## Supplementary information

**Additional file 1: Figure S1.** Impact of adjustments for aortic pulse wave velocity (PWV), estimated glomerular filtration rate (eGFR) or C-reactive protein (CRP) on multivariate adjusted left ventricular mass (LVM) beyond that predicted by stroke work (inappropriate LVM or LVM_inappr_) across octiles (Oct) of log resistin concentrations. **Figure S2.** Impact of adjustments for aortic pulse wave velocity (PWV), estimated glomerular filtration rate (eGFR) or C-reactive protein (CRP) on multivariate adjusted relations between log circulating resistin concentrations and left ventricular hypertrophy (LVH) in a community sample (*n* = 170 with LVH). **Table S1.** Bivariate relationships between circulating resistin concentrations and other factors in a community sample (*n* = 647). **Table S2.** Impact of adjustments for estimated glomerular filtration rate (eGFR), pulse wave velocity (PWV) or C-reactive protein (CRP) on the relative contribution of circulating resistin concentrations to variations in inappropriate left ventricular mass (LVM_inappr_) in a community sample (*n* = 647). **Table S3.** Impact of adjustments for estimated glomerular filtration rate (eGFR), aortic pulse wave velocity (PWV) or C-reactive protein (CRP) on the relative contribution of circulating resistin concentrations to variations in left ventricular mass index (LVMI) in a community sample (*n* = 647). **Table S4.** Circulating resistin concentrations as a determinant of inappropriate left ventricular mass (LVM_inappr_) or left ventricular mass (LVMI) in a community sample (*n* = 647).

## Data Availability

The datasets used and/or analyzed during the current study are available from the corresponding authors upon reasonable request.

## Abbreviations

BMI: Body mass index
BP: Blood pressure
CRP: C-reactive protein
DBP: Diastolic blood pressure
DM: Diabetes mellitus
eGFR: Estimated glomerular filtration rate
HbA1c: Percentage glycated hemoglobin
HOMA-IR: Homeostasis model assessment of insulin resistance
IDMS: Isotope dilution mass spectrometry
IL-1β: Interleukin-1β
IL-6: Interleukin-6
LVH: LV hypertrophy
LVM: Left ventricular mass
LVMI: LVM index
LVM_inappr_: LVM beyond that predicted by stroke work (inappropriate LVM)
PWV: Aortic pulse wave velocity
SBP: Systolic blood pressure
TNF-α: Tumor necrosis factor-α; WC: waist circumference

## References

[1] Kenchaiah S, Evans JC, Levy D, Wilson PW, Benjamin EJ, Larson MG (2002). Obesity and the risk of heart failure. New Engl J Med.

[2] Ingelsson E, Sundstrom J, Arnlov J, Zethelius B, Lind L (2005). Insulin resistance and risk of congestive heart failure. JAMA.

[3] Bahrami H, Bluemke DA, Kronmal R, Bertoni AG, Lloyd-Jones DM, Shahar E (2008). Novel metabolic risk factors for incident heart failure and their relationship with obesity: the MESA (multi-ethnic study of atherosclerosis) study. J Am Coll Cardiol.

[4] Patel L, Buckels AC, Kinghorn IJ, Murdock PR, Holbrook JD, Plumpton C (2003). Resistin is expressed in human macrophages and directly regulated by PPAR gamma activators. Biochem Biophys Res Commun.

[5] Lu SC, Shieh WY, Chen CY, Hsu SC, Chen HL (2002). Lipolysaccharide increases resistin gene expression in vivo and in vitro. FEBS Lett.

[6] Kaser S, Kaser A, Sandhofer A, Ebenbichler CF, Tilg H, Patsch JR (2003). Resistin messenger-RNA expression is increased by proinflammatory cytokines in vitro. Biochem Biophys Res Comm.

[7] Butler J, Kalogeropoulos A, Georgiopoulou V, de Rekeneire N, Rodondi N, Smith AL (2009). For the health ABC study. Serum resistin concentrations and risk of new onset heart failure in older persons. The health, aging and body composition (health ABC) study. Arterioscler Thromb Vasc Biol.

[8] Frankel DS, Vasan RS, D’Agostino RB, Benjamin EJ, Levy D, Wang TJ (2009). Resistin, adiponection, and risk of heart failure: the Framingham heart study. J Am Coll Cardiol.

[9] Zhang MH, Na B, Schiller NB, Whooley MA (2011). Associations of resistin with heart failure and mortality in patients with stable coronary heart disease: data from the heart and soul study. J Cardiac Fail.

[10] Wu X-M, Lin Y-H, Chen A, Hsu T-P, Wu Y-W, Lin H-J (2012). Prognostic significance of adipocytokines in systolic heart failure patients. Eur J Clin Investig.

[11] Takeishi Y, Niizeki T, Arimoto T, Nozaki N, Hirono O, Nitobe J (2007). Serum resistin is associated with high risk in patients with congestive heart failure-a novel link between metabolic signals and heart failure. Circ J.

[12] Muse ED, Feldman DI, Blaha MJ, Dardari ZA, Blumenthal RS, Budoff MJ (2015). The association of resistin with cardiovascular disease in the multi-ethnic study of atherosclerosis. Atherosclerosis.

[13] Norman G, Norton GR, Libhaber CD, Michel F, Majane OHI, Millen AME (2015). Independent associations between resistin and left ventricular mass and myocardial dysfunction in a community sample with prevalent obesity. Int J Cardiol.

[14] Kim M, Oh JK, Sakata S, Liang I, Park W, Hajjar RJ (2008). Role of resistin in cardiac contractility and hypertrophy. J Mol Cell Cardiol.

[15] Chemaly ER, Hadri L, Zhang S, Kim M, Kohlbrenner E, Sheng J (2011). Long-term in vivo resistin overexpression induces myocardial dysfunction and remodeling in rats. J Mol Cell Cardiol.

[16] Kang S, Chemaly ER, Hajja RJ, Lebeche D (2011). Resistin promotes cardiac hypertrophy via the AMP-activated protein kinase/mammalian target of rapamycin (AMP K/mTOR) and c-Jun N-terminal kinase/insulin receptor substrate 1 (JNK/IRS1) pathways. J Biol Chem.

[17] Schwartz DR, Briggs ER, Qatanani M, Sawaya H, Sebag IA, Picard MH (2013). Human resistin in chemotherapy-induced heart failure in humanized male mice and in women treated for breast cancer. Endocrinology.

[18] Mills KT, Hamm LL, Alper AB, Miller C, Hudaihed A, Balamuthusamy S (2013). Circulating adipocytokines and chronic kidney disease. PLoS One.

[19] Maunganidze F, Norton GR, Maseko MJ, Libhaber CD, Majane OHI, Sareli P (2013). Relationship between glomerular dysfunction and left ventricular mass independent of haemodynamic factors in a community sample. J Hypertens.

[20] Norman G, Norton GR, Gomes M, Michel F, Majane OH, Sareli P (2016). Circulating resistin concentrations are independently associated with aortic pulse wave velocity in a community sample. J Hypertens.

[21] Bello H, Norton GR, Ballim I, Libhaber CD, Sareli P, Woodiwiss AJ (2017). Contributions of aortic pulse wave velocity and backward wave pressure to variations in left ventricular mass are independent of each other. J Am Soc Hypertens.

[22] Zanoli L, Di Pino A, Terranova V, Di Marca S, Pisano M, Di Quattro R (2018). Inflammation and ventricular-vascular coupling in hypertensive patients with metabolic syndrome. Nut Metab Cardiovasc Dis.

[23] Norton GR, Majane OH, Maseko MJ, Libhaber C, Redelinghuys M, Kruger D (2012). Brachial blood pressure-independent relations between radial late systolic shoulder-derived aortic pressures and target organ changes. Hypertension.

[24] Woodiwiss AJ, Molebatsi N, Maseko MJ, Libhaber E, Libhaber C, Majane OHI (2009). Nurse-recorded auscultatory blood pressure at a single visit predicts target organ changes as well as ambulatory blood pressure. J Hypertens.

[25] Kolkenbeck-Ruh A, Woodiwiss AJ, Naran R, Sadiq E, Robinson C, Motau TH (2019). Carotid intima-media thickness, but not chronic kidney disease independently associates with non-cardiac arterial vascular events in South Africa. J Hypertens.

[26] Woodiwiss AJ, Libhaber CD, Libhaber E, Sareli P, Norton GR (2012). Relationship between on-treatment decreases in inappropriate versus absolute or indexed left ventricular mass and increases in ejection fraction in hypertension. Hypertension.

[27] Devereux RB, Alonso DR, Lutas EM, Gottlieb GJ, Campo E, Sachs I (1986). Echocardiograph assessment of left ventricular hypertrophy: comparison to necropsy findings. Am J Cardiol.

[28] Williams B, Mancia G, Spiering W, Agabiti Rosei E, Azizi M, Burnier M (2018). 2018 ESC/ESH guidelines for the management of arterial hypertension. Eur Heart J.

[29] Bell V, Sigurdsson S, Westenberg JJ, Gotal JD, Torjessen AA, Aspelund T (2015). Relations between aortic stiffness and left ventricular structure and function in older participants in the age/gene/environment susceptibility-Reykjavok study. Circ Cardiovasc Imaging.

[30] Norton GR, Peterson VR, Robinson C, Norman G, Libhaber CD, Libhaber E (2019). Independent of left ventricular mass, circulating inflammatory markers rather than pressure load are associated with concentric left ventricular remodelling. Int J Cardiol.

[31] Savage DB, Sewter CP, Klenk ES, Segal DG, Vidal-Puig A, Considine RV (2001). Resistin / Fizz3 expression in relation to obesity and peroxisome proliferator-activated receptor-gamma action in humans. Diabetes.

