# Supplementary material for: Associations between circulating resistin concentrations and left ventricular mass are not accounted for by effects on aortic stiffness or renal dysfunction
Source: BMC Cardiovasc Disord. 2020 Jan 30;20:35. doi: 10.1186/s12872-019-01319-w (PMC6993505; doi:10.1186/s12872-019-01319-w)
Supplement: Supplementary file 1 — Additional file 1: Figure S1. Impact of adjustments for aortic pulse wave velocity (PWV), estimated glomerular filtration rate (eGFR) or C-reactive protein (CRP) on multivariate adjusted left ventricular mass (LVM) beyond that predicted by stroke work (inappropriate LVM or LVMinappr) across octiles (Oct) of log resistin concentrations. Figure S2. Impact of adjustments for aortic pulse wave velocity (PWV), estimated glomerular filtration rate (eGFR) or C-reactive protein (CRP) on multivariate adjusted relations between log circulating resistin concentrations and left ventricular hypertrophy (LVH) in a community sample (n = 170 with LVH). Table S1. Bivariate relationships between circulating resistin concentrations and other factors in a community sample (n = 647). Table S2. Impact of adjustments for estimated glomerular filtration rate (eGFR), pulse wave velocity (PWV) or C-reactive protein (CRP) on the relative contribution of circulating resistin concentrations to variations in inappropriate left ventricular mass (LVMinappr) in a community sample (n = 647). Table S3. Impact of adjustments for estimated glomerular filtration rate (eGFR), aortic pulse wave velocity (PWV) or C-reactive protein (CRP) on the relative contribution of circulating resistin concentrations to variations in left ventricular mass index (LVMI) in a community sample (n = 647). Table S4. Circulating resistin concentrations as a determinant of inappropriate left ventricular mass (LVMinappr) or left ventricular mass (LVMI) in a community sample (n = 647). [file 12872_2019_1319_MOESM1_ESM.doc]

**On-line supplement**

**Associations Between Circulating Resistin Concentrations and Left Ventricular Mass are Not Accounted for by Effects on Aortic Stiffness or Renal Dysfunction.**

Glenda Norman, Gavin R Norton, Vernice Peterson, Monica Gomes, Carlos D Libhaber, Pinhas Sareli, Angela J Woodiwiss.

Cardiovascular Pathophysiology and Genomics Research Unit, School of Physiology, Faculty of Health Sciences, University of the Witwatersrand, Johannesburg, South Africa.

**Running title**: Resistin and LV mass.

**Conflict of interest:** None of the authors have any conflicts of interest to declare

GN, GRN and AJW contributed equally to this work.

This work was supported by the Medical Research Council of South Africa, the Circulatory Disorders Research Trust, the University Research Council of the University of the Witwatersrand, and the National Research Foundation of South Africa.

Correspondence and reprint requests: Angela J Woodiwiss and Gavin R Norton: Cardiovascular Pathophysiology and Genomics Research Unit, School of Physiology, University of the Witwatersrand Medical School, 7 York Road, Parktown, 2193, Johannesburg, South Africa. Tel: +27 11 717 2363, e-mail: [angela.woodiwiss@wits.ac.za](mailto:angela.woodiwiss@wits.ac.za) and [gavin.norton@wits.ac.za](mailto:gavin.norton@wits.ac.za)

**Figure S1.** Impact of adjustments for aortic pulse wave velocity (PWV), estimated glomerular filtration rate (eGFR) or C-reactive protein (CRP) on multivariate adjusted left ventricular mass (LVM) beyond that predicted by stroke work (inappropriate LVM or LVM_inappr_) across octiles (Oct) of log resistin concentrations. Additional adjustments are for age, sex, waist circumference, office systolic blood pressure, treatment for hypertension, diabetes mellitus or an HbA1c>6.5%, regular smoking, and regular alcohol intake. *p<0.05, **p<0.005 vs octile 1; ^†^p<0.05, ^††^p<0.005 vs octile 2; ^‡^p<0.05, ^‡‡^p<0.005 vs octiles 3 and 4; #p<0.05 vs octile 5.

**Figure S2.** Impact of adjustments for aortic pulse wave velocity (PWV), estimated glomerular filtration rate (eGFR) or C-reactive protein (CRP) on multivariate adjusted relations between log circulating resistin concentrations and left ventricular hypertrophy (LVH) in a community sample (n=170 with LVH). Additional adjustments are for age, sex, waist circumference, systolic blood pressure, treatment for hypertension, diabetes mellitus or an HbA1c>6.5%, regular smoking, and regular alcohol intake.

Figure S1


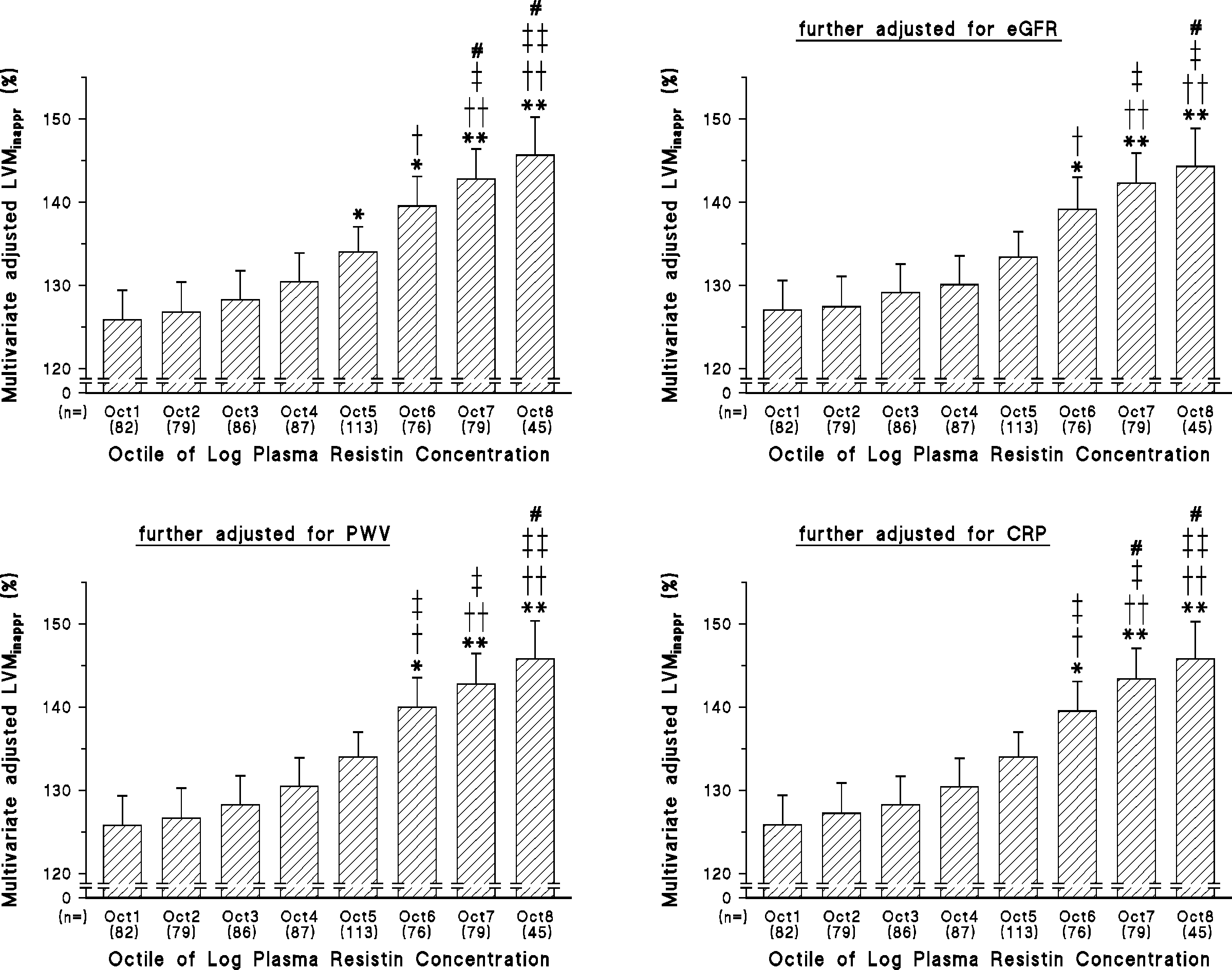


Figure S2


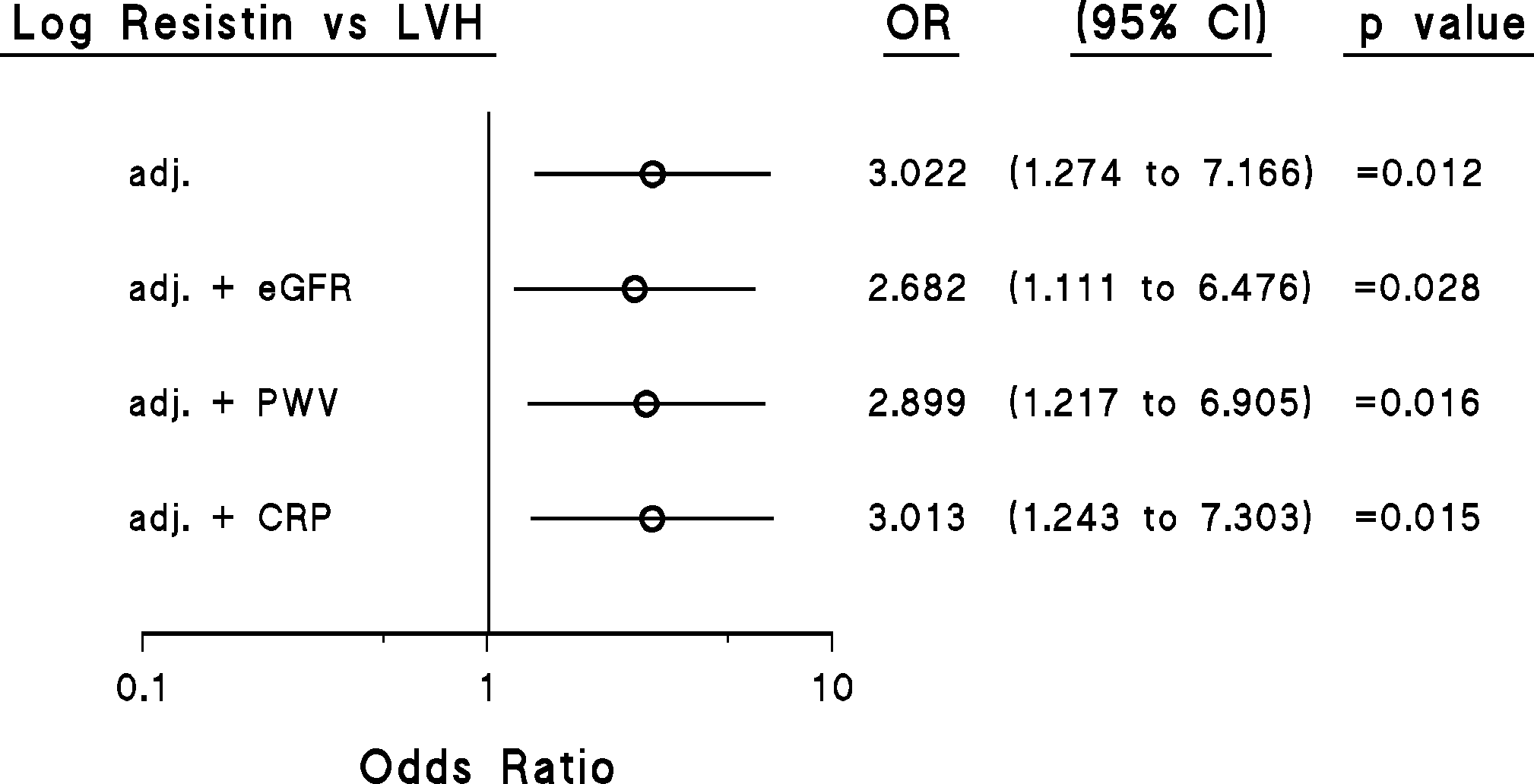


**Table S1**. Bivariate relationships between circulating resistin concentrations and other factors in a community sample (n=647).

Log resistin vs Pearson’s r p value

___________________________________________________________________________

Age 0.141 =0.0003

BMI 0.127 =0.0012

Waist circumference 0.115 =0.0036

SBP 0.092 =0.020

eGFR -0.246 <0.0001

PWV 0.154 <0.0001

CRP 0.248 <0.0001

HOMA-IR 0.024 =0.54

LVMI 0.157 <0.0001

LVM_inappr_ 0.181 <0.0001

Male gender 0.108 =0.0057

Regular smoking 0.049 =0.21

Regular alcohol consumption 0.038 =0.34

Diabetes mellitus or HbA1c >6.5% 0.070 =0.07

Treatment for hypertension 0.116 =0.0010

__________________________________________________________

BMI, body mass index; SBP, office systolic blood pressure; eGFR, estimated glomerular filtration rate; PWV, pulse wave velocity; CRP, C-reactive protein; HOMA-IR, homeostasis model of insulin resistance; LVMI, left ventricular mass index; LVM_inappr_, inappropriate left ventricular mass.

**Table S2**. Impact of adjustments for estimated glomerular filtration rate (eGFR), pulse wave velocity (PWV) or C-reactive protein (CRP) on the relative contribution of circulating resistin concentrations to variations in inappropriate left ventricular mass (LVM_inappr_) in a community sample (n=647).

Models with→ * * + eGFR * + PWV * + CRP

β-coeff ±SEM p value β-coeff±SEM p value β-coeff±SEM p value β-coeff±SEM p value

____________________________________________________________________________________________________________________

Age -0.075±0.053 =0.16 -0.156±0.062 =0.012 -0.068±0.057 =0.23 -0.076±0.053 =0.15

Male gender 0.128±0.042 =0.0026 0.129±0.040 =0.0013 0.128±0.042 =0.0027 0.130±0.043 =0.0027

Waist circumference 0.256±0.047 <0.0001 0.260±0.046 <0.0001 0.257±0.047 <0.0001 0.250±0.050 <0.0001

SBP -0.120±0.044 =0.0062 -0.129±0.043 =0.0031 -0.115±0.045 =0.012 -0.119±0.044 =0.0066

Log resistin 0.158±0.037 <0.0001 0.138±0.038 =0.0003 0.159±0.038 <0.0001 0.157±0.038 <0.0001

eGFR or PWV or CRP - - -0.133±0.053 =0.013 -0.016±0.050 =0.75 0.009±0.045 =0.84

HOMA-IR 0.100±0.039 =0.011 0.082±0.040 =0.039 0.100±0.039 =0.011 0.104±0.039 =0.0083

___________________________________________________________________________________________________________________

β-coeff, standardized β-coefficient; SBP, office systolic blood pressure; HOMA-IR, homeostasis model of insulin resistance. *Additional factors included in the stepwise regression models include regular smoking, regular alcohol intake, treatment for hypertension and diabetes mellitus or an HbA1c>6.5%.

**Table S3**. Impact of adjustments for estimated glomerular filtration rate (eGFR), aortic pulse wave velocity (PWV) or C-reactive protein (CRP) on the relative contribution of circulating resistin concentrations to variations in left ventricular mass index (LVMI) in a community sample (n=647).

Models with→ * * + eGFR * + PWV * + CRP

β-coeff ±SEM p value β-coeff±SEM p value β-coeff±SEM p value β-coeff±SEM p value

___________________________________________________________________________________________________________________

Age 0.105±0.051 =0.038 0.045±0.059 =0.45 0.069±0.055 =0.21 0.106±0.051 =0.039

Male gender 0.011±0.041 =0.78 0.012±0.040 =0.76 0.012±0.041 =0.76 0.013±0.041 =0.75

Waist circumference 0.200±0.045 <0.0001 0.203±0.045 <0.0001 0.202±0.045 <0.0001 0.200±0.048 <0.0001

SBP 0.207±0.042 <0.0001 0.200±0.042 <0.0001 0.186±0.043 <0.0001 0.207±0.042 <0.0001

Log resistin 0.095±0.036 =0.0081 0.081±0.037 =0.028 0.091±0.036 =0.012 0.096±0.037 =0.0093

eGFR or PWV or CRP - - -0.099±0.051 =0.053 0.084±0.048 =0.083 -0.002±0.043 =0.96

HOMA-IR 0.083±0.037 =0.028 0.069±0.038 =0.069 0.078±0.037 =0.037 0.084±0.038 =0.026

___________________________________________________________________________________________________________________

β-coeff, standardized β-coefficient; SBP, office systolic blood pressure; HOMA-IR, homeostasis model of insulin resistance. *Additional factors included in the stepwise regression models include regular smoking, regular alcohol intake, treatment for hypertension and diabetes mellitus or an HbA1c>6.5%.

**Table S4**. Circulating resistin concentrations as a determinant of inappropriate left ventricular mass (LVM_inappr_) or left ventricular mass (LVMI) in a community sample (n=647).

LVM_inappr_ LVMI

Models with→ * *

Partial r^2^ p value Partial r^2^ p value

_________________________________________________________________________________________

Waist circumference 0.079 <0.0001 Waist circumference 0.136 <0.0001

SBP 0.031 <0.0001 SBP 0.053 <0.0001

Log resistin 0.030 <0.0001 Age 0.012 =0.0010

Male gender 0.012 =0.0016 Log resistin 0.010 =0.0022

HOMA-IR 0.008 =0.0089 HOMA-IR 0.007 =0.010

Age 0.000 =0.37 Male gender 0.0001 =0.82

Model r^2^ 0.164 Model r^2^ 0.222

_________________________________________________________________________________________________________________

SBP, office systolic blood pressure; HOMA-IR, homeostasis model of insulin resistance. *Additional factors included in the stepwise regression models include regular smoking, regular alcohol intake, treatment for hypertension and diabetes mellitus or an HbA1c>6.5%.
